# The Impact of the COVID-19 Lockdown on Weight Loss and Body Composition in Subjects with Overweight and Obesity Participating in a Nationwide Weight-Loss Program: Impact of a Remote Consultation Follow-Up—The CO-RNPC Study

**DOI:** 10.3390/nu13072152

**Published:** 2021-06-23

**Authors:** Sébastien Bailly, Odile Fabre, Rémy Legrand, Laurent Pantagis, Monique Mendelson, Robin Terrail, Renaud Tamisier, Arne Astrup, Karine Clément, Jean-Louis Pépin

**Affiliations:** 1 HP2 Laboratory, Grenoble Alpes University, INSERM U1300 and Grenoble Alpes University Hospital, 38000 Grenoble, France; mmendelson@chu-grenoble.fr (M.M.); rterrail@chu-grenoble.fr (R.T.); rtamisier@chu-grenoble.fr (R.T.); JPepin@chu-grenoble.fr (J.-L.P.); 2 Groupe Éthique et Santé. Actiburo 1, Bâtiment A—100 Chemin de l’Aumône Vieille, 13400 Aubagne, France; odile.fabre@groupethiquetsante.com (O.F.); remy.legrand@groupethiquetsante.com (R.L.); laurent.pantagis@groupethiquetsante.com (L.P.); 3 Department of Nutrition, Exercise and Sports, University of Copenhagen, 1165 Copenhagen, Denmark; ara@novo.dk; 4 Nutrition Department, Assistance Publique-Hôpitaux de Paris, Pitié-Salpêtrière Hospital, 75013 Paris, France; karine.clement2@gmail.com; 5 Nutrition and Obesities: Systemic Approaches (NutriOmics) Research Unit, Sorbonne Université, INSERM, 75013 Paris, France

**Keywords:** COVID-19 pandemic, total lockdown, weight loss program, remote consultations

## Abstract

The aim of this study was to assess the impact of the nationwide total lockdown (LD) in France on weight loss and body composition modifications in subjects participating in a weight loss program and to evaluate the impact of remote consultations on participants’ adherence to the weight loss program. The CO-RNPC study was a prospective multicentre cohort study including participants undergoing a two to six months program. The rate of weight loss in kg/week was computed before (15 days), during (99 days) and after LD (15 days). In the 1550 completing participants, body weight decreased from 87.1 kg [IQR 77.0; 100.2] to 82.3 kg [72.1; 94.3] resulting in a difference of −4.79 kg [−4.48; −5.10] (*p* < 0.01), with a corresponding reduction in waist circumference by 4 cm ([0; 9], *p* < 0.01). The median weight loss was 4.4 kg [0.5; 9.4] in those who used remote consultations, and 1.4 kg [0.8; 5.7] in the no remote consultation group (*p* < 0.01). In this large prospective cohort, we observed that the rate of weight loss was reduced during LD. This reduction was counterbalanced in participants involved in a remote consultation follow-up with a dose-effect response based on the number of remote consultations.

## 1. Introduction

The COVID-19 pandemic has forced countries around the world to develop various strategies to slow down and contain the spread of the SARS-CoV-2. In France, a nationwide total lockdown (LD) was implemented on 17 March 2020 to reduce the burden on intensive care units in the two main affected regions of France, and to prevent the spread of an uncontrolled epidemic to other regions [1,2]. During the LD, people were asked to stay at home and were only allowed to leave their homes to buy essential goods such as food or medical supplies, or for legal obligations. Individual physical activity was allowed within a delimited perimeter (one kilometer) for a maximum of one hour daily [3]. All working activities were suspended or turned into remote working, except for essential services (health-care workers, food supply and sale, police, etc.).

The imposed LD has had a significant impact on habitual lifestyle in the general population. Overall, there was a decrease in physical activity levels, an increase in sedentary time associated with an alteration of eating behaviors [4], and an objectively measured reduction in the number of steps per day of up to a 50% [3]. Furthermore, the LD period lead to overeating, increased screen time, and sleep disturbances [5,6,7]. Social isolation also has the potential to worsen lifestyle behaviors by increasing sedentary time, decreasing outdoor time and physical activity, which in turn favors weight gain [8]. Such lifestyle modifications (eating habits and physical activity levels) were exacerbated in people with obesity [9,10]. Worldwide, 60% of the population are overweight, of which 25% are obese. Obesity is recognized as a major health problem with multiple consequences for public health [11]. Obesity is associated with an increased susceptibility to infections [12], and there is an increased risk of comorbidities and mortality associated with increased BMI [13]. In the current context of the COVID-19 pandemic, being overweight and obese is a major factor associated with severe forms of COVID-19 infection [14], and there is a high prevalence of patients with obesity with an increased risk of complications and mortality in intensive care units [15,16]. The specificities of the persons with overweight or obesity highlight the importance of sustaining and protecting weight loss interventions during this pandemic period [16,17].

However, there is a lack of data on the impact of LD on populations who were already involved in a weight loss program prior to LD. This unprecedented context has accelerated the development of remote consultations to ensure adherence to the proposed weight loss program and to maintain weight loss trajectories. The RNPC^®^ nationwide program (79 centres for Rééducation Nutritionnelle Psycho-Comportementale, i.e., Nutritional Psycho-Behavioural Rehabilitation) [18] was prospectively evaluated in terms of weight loss and body composition changes before, during, and after LD.

The main objective of the CO-RNPC study was to assess the impact of the Covid-19 LD on the rate of weight loss achieved by individuals with overweight or obesity already participating in a weight loss program. The secondary objective was to evaluate the impact of LD on body composition, and to address whether participants’ adherence to remote consultations had an impact on weight loss success.

## 2. Materials and Methods

### 2.1. Study Design

**Figure 1:**
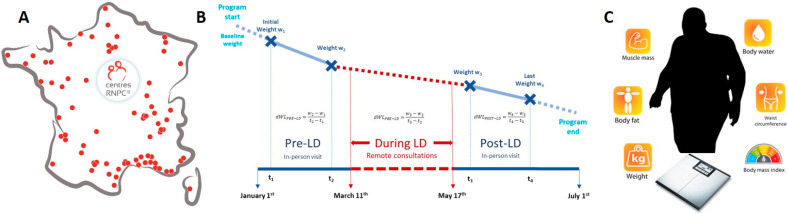
Study design and outcomes assessed in participants in a multicenter nationwide weight loss program. (**A**) Geographical distribution of the RNPC^®^ centers in France (**B**) Study design: data were prospectively collected at two time-points before and after the nationwide total lockdown (LD) due to Covid19 in France, during in-person appointments with a RNPC^®^ dietician. Remote consultations with the RNPC^®^ dietician were conducted by phone during the LD period. Median period durations between two measures: before lockdown (t_2_-t_1_): 15 days [15; 17], during LD (t_3_-t_2_): 99 days [92; 106], after LD (t_4_-t_3_): 15 days [15; 21]—dWL: delta weight loss (dWL) for details regarding calculation of dWL, see “Outcomes and definitions”. (**C**) Data collection: at a face-to-face consultation with a dietician, measurements made using an impedance scale (the same Beurer BG42 calibrated bioelectrical device) for weight, muscle mass, fat mass and body water. Waist circumference was measured to the nearest cm at the natural waist. Body mass index was calculated by dividing the weight in kilos by the height squared.

The CO-RNPC study was a prospective multicentre cohort conducted in the RNPC^®^ centres in France (*n* = 79; Figure 1). The weight loss program has been extensively presented elsewhere [18]. Briefly, the RNPC^®^ program (for Rééducation Nutritionnelle Psycho-Comportementale, i.e., Nutritional Psycho-Behavioural Rehabilitation) is a three-stage weight loss program performed in 79 centres distributed across France. Participants are referred to the RNPC^®^ program by their clinician, essentially general practitioners, to co-determine an objective of weight loss. The two stages of the program are: (1) a weight loss phase during which rapid weight loss is achieved (two to six months) and (2) a stabilization phase during which energy intake is gradually increased with a duration depending on the weight loss phase (i.e., 1 week per kg lost during the initial phase). The program is followed-up with a maintenance phase during which energy balance is achieved. In the maintenance period participants can return to the program whenever necessary. Face-to-face consultation with RNPC^®^ dieticians are scheduled once every two weeks during the first two stages (weight loss and stabilization), and then become optional during the maintenance phase. As participants were beginning their program, face-to-face consultations before LD were mandatory. Ethical approval for the study was issued by the CPP Ile de France III under approval number 2020-A01210–39.

### 2.2. Remote Consultations

In the exceptional circumstances of total LD in France due to the Covid-19 pandemic, the participant tracking software (RNPC PILOT) was optimized to include a module dedicated to remote consultations and regular monitoring. Dieticians were advised to phone participants every other week for a 20-min consultation to maintain follow-up. Participants could thus benefit from the professional skills of their dietitian without leaving home. As with face-to-face consultations, dieticians provided counselling to help participants manage eating behaviour and keep them motivated to follow the program to lose weight and/or maintain weight loss. During follow-up, participants included in the study were identified according to whether they engaged in the remote consultation services or not. The objectives for remote consultations were: (1) to assess if there was a difference in weight loss according to the choice of benefiting from remote consultations or not, and (2) to identify if there was a dose-response according to the number of remote consultations observed by participants.

### 2.3. Data Collection and Measures

Data were systematically and homogeneously collected in each RNPC^®^ centre by using the same electronic medical record (RNPC PILOT) for participant characteristics (age, gender), status of tobacco consumption, anthropometric measures (weight, body mass index, waist circumference, percent of fat mass, muscle mass and body water). Body weight, body water, muscle and fat percentages were measured at follow-up visits before and after LD with a calibrated bioelectrical impedance scale (BG42, Beurer, Ulm, Germany). Height was measured at the first visit to the nearest cm using a height gauge. Waist circumference (WC) was measured to the midpoint between the lower border of the rib cage and the iliac crest. For participants with abdominal adiposity with no visible natural waist, the measurement was taken approximately 2–5 cm above the navel. Main comorbidities, drug treatments from which chronic comorbidities were identified (diabetes, hypertension), and the number of remote consultations were also registered.

### 2.4. Population

In this study, to correctly assess the specific impact of LD in a well-defined population, only participants who were in the weight loss phase (Phase 1) before and after the total LD in France (from 11 March to 17 May 2020), with at least two objective face-to-face assessments before and after LD were considered. Participants with only one weight measure before or after LD, participants aged under 18 years or with a body mass index (BMI) under 25 kg/m^2^ were excluded.

### 2.5. Outcomes and Definitions

The rate of weight loss (delta weight loss dWL in kg/week) before LD was the ratio of the difference between the two last measures of body weight before LD on the number of weeks between the two measures. The dWL during LD was the ratio of the difference between the last measure before LD and the first measure after LD on the time between these two measures. The dWL after LD was the ratio of the difference between the first measure of body weight after LD minus the last measure of weight loss collected in this study on the number of weeks between the two measures. Secondary outcome criteria were calculated in the same way for waist circumference, percentage of fat mass, muscle mass, and changes in water mass.

### 2.6. Statistical Methods

Variables were described as median and interquartile range for quantitative variables and number and percentage for qualitative variables. Comparison of body weight and criteria of body composition between baseline and last measure of the follow-up were performed by using nonparametric paired Wilcoxon rank tests. To assess the evolution of dWL over time (before, during and after LD), a generalised linear mixed model was used with a random effect for participants. The same model was built for the evolution of body composition parameters. All models were adjusted for the first value of the measured parameter (weight, waist circumference, percent of fat mass, muscle mass or body water), age, gender and use of teleconsultations during LD. An interaction term was tested between the time of the study and the number of remote consultations. As it was not significant it was not retained in the final model. Statistical analyses were performed using SAS v. 9.4 (SAS Institute, Cary, NC). A *p*-value threshold of 0.05 was considered significant.

## 3. Results

### 3.1. Study Population

From 3697 participants in phase 1 of the RNPC program, 1550 participants met the inclusion criteria of the study. They were predominantly women (80.4%), with a median age of 54 years interquartile range (IQR): [46; 62], a median initial weight of 87 kg [77; 100] and a median BMI of 31.9 [28.8; 35.6] kg/m^2^. The median period durations between two measures were: before LD: 15 days [15; 17], during LD: 99 days [92; 106], after LD: 15 days [15; 21].

### 3.2. Changes in Rate of Weight Loss during COVID-19 Total Lockdown

#### Primary Outcome: Rate of Weight Loss (dWL)

**Figure 2:**
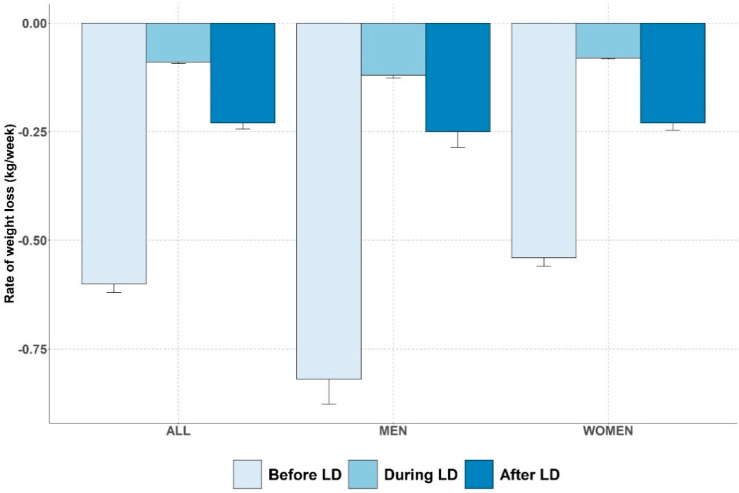
Rates of weight loss before, during and after COVID19 lockdown periods depicted for overall population and per gender. LD: lockdown.

Before LD, body weight decreased from 87.1 kg [77.0; 100.2] to 86.2 kg [76.0; 98.7] resulting in a median decrease of 1.3% [0.3; 2.2] of initial body weight during a median duration of 15 days [15; 17] (Table 1, Figure 2).

During the LD period, body weight decreased from 86.2 kg [76.0; 98.7] to 83.3 kg [73.5; 95.1], resulting in a median decrease of 2.9% [0.6; 7.2] of initial body weight, during a median duration of 99 days [92; 106].

Finally, there is a significant difference in the rate of weight loss in kg/week before LD (0.5 kg/week [0.1; 1]) compared to during LD (0.1 kg/week [0; 0.1]), *p* < 0.01.

After LD, there was a decrease in body weight from 83.3 kg [73.5; 95.1] to 82.9 kg [72.9; 94.7] corresponding in a median decrease of 0.6% [0.3; 1.5] of initial body weight during a median duration of 15 days [15; 21]. The rate of weight change after LD was 0.2 kg/week [−0.1; 0.6] and was significantly higher compared to the rate of weight change during LD (*p* < 0.01) (Figure 2).

The dWL were significantly different between men and women. Men exhibited higher baseline weight and a greater weight loss during the study period than women.

### 3.3. Rate of Change in Body Composition during COVID-19 Total Lockdown

**Figure 3:**
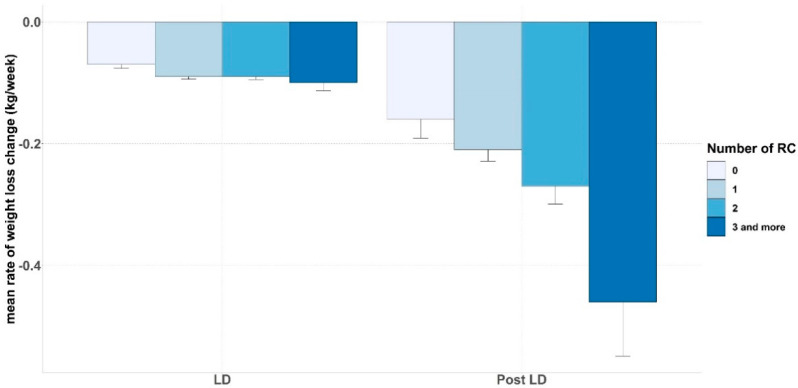
Rates of weight loss according to the number of remote consultation during and after the COVID19 lockdown periods. LD: lockdown; graph legend: number of remote consultation.

Before LD, waist circumference decreased from 102 cm [93; 112] to 101 cm [92; 111] resulting in a median decrease of 1 cm [0; 2] during a median duration of 15 days [15; 17]. During the LD period, waist circumference decreased from 101 cm [92; 111] to 98 cm [90; 107] during a median duration of 99 days [92; 106] (Table S1, Figure 3).

There was a significant difference in the rate of change in waist circumference, which was higher before LD (−0.5 cm/week [−1; 0]) compared to the LD period (−0.07 cm/week [−0.15; 0]) (*p* < 0.01).

The rate of change in fat mass was −0.15% per week [−0.35; 0] during the pre-LD period compared to −0.03% per week [−0.05; 0] during the LD period, and −0.07% per week [−0.24; 0.05] in the post LD period (*p* < 0.01).

The same trends applied for body water, and there was no significant trend in the rate of change in muscle mass (Supplementary Table S1, Figure S1). Difference according men and women are presented in Supplementary Tables S2 and S3.

### 3.4. Impact of Remote Consultation Follow-up

Most included subjects chose remote consultations during LD (*n* = 1285, 82.9%), with a median number of one remote consultation during LD IQR: [1; 2]. The sub-group of subjects who chose to use remote consultations had a higher BMI, fat mass percentage and waist circumference at baseline than those who did not (Table 2). The median weight loss in the group with remote consultation was −4.4 kg [−9.4; −0.5] and significantly higher compared to the group without remote consultation −1.4 kg [−5.7; 0.8]. The rates of weight loss were significantly different between groups in pre-LD (−0.4 [−0.8; −0.1] vs. during LD −0.6 [−1; −0.2], *p* < 0.01) and post-LD (−0.2 [−0.4; 0.2] vs. during LD −0.2 [−0.6; 0.1], *p* < 0.01).

Weight and fat loss were improved by remote consultations in a dose-response relationship according to the number of remote consultations. For weight loss an additional decrease of 0.06 kg/week for one remote consultation was seen, and a weight loss of 0.18 kg/week for more than three remote consultations (*p* < 0.01) was observed (Supplementary Table S4 and Figure S3). For percentage of fat mass an additional decrease of 0.02%/week was seen for one remote consultation, and a decrease of 0.28%/week was found for three or more remote consultations (*p* = 0.04). An additional increase in muscle mass of 0.07%/week was found for one remote consultation, and an increase of 0.40%/week was found for three or more remote consultations (*p* < 0.01). The number of remote consultations did not have a significant impact on the decrease in waist circumference (*p* = 0.51).

## 4. Discussion

In this large prospective cohort of individuals undergoing a weight loss program, we found that the rate of weight loss was reduced during LD compared to before and after LD. However, the adverse effect of LD was attenuated in participants who chose to take advantage of remote consultation, with a dose-effect response based on the number of remote consultations. The impact of LD was similar for effect on waist circumference, fat mass and percent of body water.

Although there was a decrease in the weight loss trajectory during LD, we found that participants included in the RNPC^®^ program continued to demonstrate a median decrease of 2.4 kg [0.5; 6.4] and a median decrease in waist circumference of 2 cm [; 6]. There are a limited number of studies objectively and prospectively reporting how weight loss programs are affected by LD. The overall weight loss observed for participants in the RNPC^®^ program is in contrast with what has been reported for the general population. In a study including patients with obesity, the LD period was associated with a weight gain of over 1.5 kg [10]. The COVID UK data reported an average increase in body weight from 0.78 kg to 3 kg across the country [19]. In France, the IFOP survey found that 57% of the French population reported a mean weight gain of 2.5 kg [20].

Compared to these observations, it is interesting that the participants in our cohort, who were enrolled in a weight loss program, still achieved significant weight loss during LD. This was significantly improved by the initiation of remote consultations, which were aimed at providing support from an experienced dietician and maintaining adherence to the weight loss program. However, it is important to interpret these results with caution because it is possible that participants who chose to benefit from remote consultations presented different characteristics with regards to their behavioral compliance than those who chose not to. The results demonstrate the efficacy and feasibility of remote monitoring to support weight loss programs even in a difficult pandemic situation. This suggests potential for systematically include remote consultation in weight loss programs. The importance of the psycho-behavioural approach in weight loss programs is well documented [21] and these digital tools can help to achieve the individualization of patient management [22]. In fact, a number of studies support the effectiveness of remote interventions using e-Health for the prevention and treatment of overweight and obesity in adults. A recent meta-analysis demonstrated significantly greater weight loss in eHealth weight interventions compared to control (i.e., no intervention) or minimal interventions (i.e., written materials) in adults [23]. Another meta-analysis confirmed the effectiveness of telemedicine on decreasing body mass index in adults with obesity, hypertension and/or type 2 diabetes [24].

The importance of the behavioural approach in weight loss programs has been reported previously in a systematic review [25]. The RNPC^®^ program is based on the individualisation of follow-up during the program, using face-to-face consultation with a dietician at least once every two weeks. The impact of LD on body weight was counteracted by the development and implementation of remote consultations, which proved to be effective in preventing the decrease in weight loss trajectory, as was observed in participants who did not use the remote consultations. This data regarding the relationship between the number of remote consultations and the slope of weight loss is unique. A next step would be to determine the best combination of face-to-face and remote consultations in terms of cost-effectiveness [26].

The present study has several strengths. To our knowledge, it is the first prospective study based on a large sample of subjects who followed the same weight loss program based in a well-defined population. Data are robust and based on objective measurements, as compared to many studies using self-reported data (i.e., surveys), such as the ECLB-COVID19 International Online Survey [4]. To our knowledge, there are few studies that report objective measures of weight during LD for overweight and obese subjects [10,27,28]. This study allows a more accurate assessment of the impact of a structured and individualized weight loss program, and showed an association between remote consultations and weight loss trajectories during the COVID-19 outbreak.

Nevertheless, the study does have limitations. Firstly, there was no objective assessment of participant’s daily physical activity levels and food intake during the study. Secondly, only the RNPC^®^ weight loss program was assessed, and it was not compared to other weight loss programs, thus limiting generalization of the findings. Thirdly, only patients with two measures before and after LD were considered in order to assess the impact of LD on weight loss, thereby excluding patients who stopped the program during LD. Finally, the association between the number of remote consultations and weight loss success may have two causal pathways. We suggest that it is the remote consultations that enhanced the weight loss success, but we cannot exclude the possibility that participants who chose to use the remote consultations are more controlled, more motivated and have more adherent personalities, in which case the number of remote consultations is simply a marker of their personality traits. Two approaches might be performed to strengthen these results: (1) an observational study aiming to compare weight loss trajectories in a period without LD to establish the slowing of weight loss during the program in a non-pandemic situation and (2) a randomized trial would be desirable to evaluate causality for demonstrating impact of remote consultations.

## 5. Conclusions

In the CO-RNPC study we found that during the exceptional circumstances related to the COVID-19 pandemic (i.e., lockdown), participants enrolled in a weight loss program continued their weight loss trajectory, in contrast to an overall increase in body weight observed nationwide in France, and that this weight loss was improved in participants who chose to use the remote consultations. These results highlight the need to develop individualized programs in vulnerable populations to maintain weight loss trajectories during challenging societal periods. Further studies are needed to identify different profiles of weight loss trajectory according to varying lifestyle behaviours during LD. This can be achieved by examining different samples of subjects answering qualitative surveys of their lifestyle behaviours in terms of physical activity, eating habits, mood disorders and sleep.

## Figures and Tables

**Table 1:** Description of weight loss and rate of weight loss before, during and after COVID19 lockdown periods.

	Period	Initial Weight	Final Weight	Delta Weight (kg)	Delta Weight (%)	*p*-Value *
All (*n* = 1550)	Before Lockdown	87.1 [77.0; 100.2]	86.2 [76.0; 98.7]	−1.1 [−2.0; −0.3]	−1.3 [−2.2; −0.3]	<0.01
	Lockdown	86.2 [76.0; 98.7]	83.3 [73.5; 95.1]	−2.4 [−6.4; 0.5]	−2.9 [−7.2; 0.6]	<0.01
	After lockdown	83.3 [73.5; 95.1]	82.9 [72.9; 94.7]	−0.5 [−1.2; 0.2]	−0.6 [−1.5; 0.3]	<0.01
Women (*n* = 1246)	Before Lockdown	105.9 [96.0; 114.6]	104.0 [95.2; 113.0]	−1.5 [−2.8; −0.3]	−1.4 [−2.6; −0.3]	<0.01
	Lockdown	104.0 [95.2; 113.0]	99.1 [91.6; 108.3]	−3.2 [−8.4; 0.4]	−3.2 [−7.7; 0.3]	<0.01
	After lockdown	99.1 [91.6; 108.3]	98.6 [91.1; 107.2]	−0.5 [−1.6; 0.3]	−0.5 [−1.5; 0.3]	<0.01
Men (*n* = 304)	Before Lockdown	83.5 [75.2; 94.3]	82.5 [74.2; 92.7]	−1.1 [−1.9; −0.3]	−1.3 [−2.2; −0.4]	<0.01
	Lockdown	82.5 [74.2; 92.7]	79.2 [71.5; 89.5]	−2.3 [−6.0; 0.5]	−2.9 [−6.9; 0.6]	<0.01
	After lockdown	79.2 [71.5; 89.5]	78.8 [71.0; 88.7]	−0.5 [−1.2; 0.2]	−0.6 [−1.4; 0.3]	<0.01

Median period durations between two measures: before lockdown: 15 days [15; 17], during LD: 99 days [92; 106], after LD: 15 days [15; 21]. Values are presented in median and interquartile range. * All *p* values were results of paired t-test comparing initial and final weights.

**Table 2:** Subject characteristics.

	No Remote Consultation (*n* = 265)	Remote Consultation (*n* = 1285)	*p* Value
Sex (female)	220 (79.1)	1076 (81.4)	0.38
Age (year)	54 [46; 61]	54 [45; 63]	0.31
Tobacco consumption	32 (11.5)	150 (11.3)	0.94
Menopause	104 (37.4)	534 (40.4)	0.36
Sleep apnea	31 (11.2)	240 (18.2)	<0.01
Arthrosis	87 (31.3)	483 (36.5)	0.10
Venous disease	38 (13.7)	216 (16.3)	0.27
Diabetes	8 (2.9)	54 (4.1)	0.34
Hyper-triglyceridemia	0 (0)	7 (0.5)	0.22
Initial body mass index (kg/m^2^)	33.2 [29.4; 36.8]	34.2 [30.8; 38.3]	<0.01
Initial fat mass (%)	41 [36.8; 45]	42.3 [38.9; 46.2]	<0.01
Initial muscle mass (%)	29.3 [27.4; 31.6]	29.1 [26.9; 32.2]	0.82
Initial percentage of body water (%)	43 [40.1; 45.9]	42 [39.2; 44.5]	<0.01
Initial waist circumference (cm)	106 [96; 119]	110 [100; 120]	0.02

Values are actual number of subjects and percent for qualitative variables and median and interquartile range. Remote consultation group: subjects who were contacted by the RNPC^®^ dieticians by phone. No remote consultation group: subjects who did not use the service.

## Data Availability

The data presented in this study are available on request from the corresponding author. The data are not publicly available due to GRPD.

## Supplementary Materials

The following are available online at https://www.mdpi.com/article/10.3390/nu13072152/s1, Figure S1: Rates of change in body composition before, during and after COVID19 lockdown, Table S1: Description of changes in body composition and rate of change before, during and after COVID19 lockdown periods, Table S2: Description of changes in body composition and rate of change before, during and after COVID19 lockdown periods for women (*N* = 1246) Table S3: Description of changes in body composition and rate of change before, during and after COVID19 lockdown periods for men (*N* = 304) Table S4: Comparison of weight loss and rate of weight loss according to participation in remote consultation follow-up.

## References

[1] Cauchemez S., Kiem C.T., Paireau J., Rolland P., Fontanet A. (2020). Lockdown impact on covid-19 epidemics in regions across metropolitan france. Lancet.

[2] Salje H., Tran Kiem C., Lefrancq N., Courtejoie N., Bosetti P., Paireau J., Andronico A., Hoze N., Richet J., Dubost C.L. (2020). Estimating the burden of sars-cov-2 in france. Science.

[3] Pepin J.L., Bruno R.M., Yang R.Y., Vercamer V., Jouhaud P., Escourrou P., Boutouyrie P. (2020). Wearable activity trackers for monitoring adherence to home confinement during the covid-19 pandemic worldwide: Data aggregation and analysis. J. Med. Internet Res..

[4] Ammar A., Brach M., Trabelsi K., Chtourou H., Boukhris O., Masmoudi L., Bouaziz B., Bentlage E., How D., Ahmed M. (2020). Effects of covid-19 home confinement on eating behaviour and physical activity: Results of the eclb-covid19 international online survey. Nutrients.

[5] Sher L. (2020). Covid-19, anxiety, sleep disturbances and suicide. Sleep Med..

[6] Huang Y., Zhao N. (2020). Generalized anxiety disorder, depressive symptoms and sleep quality during covid-19 outbreak in china: A web-based cross-sectional survey. Psychiatry Res..

[7] Voitsidis P., Gliatas I., Bairachtari V., Papadopoulou K., Papageorgiou G., Parlapani E., Syngelakis M., Holeva V., Diakogiannis I. (2020). Insomnia during the covid-19 pandemic in a greek population. Psychiatry Res..

[8] Balanza-Martinez V., Atienza-Carbonell B., Kapczinski F., De Boni R.B. (2020). Lifestyle behaviours during the covid-19-time to connect. Acta Psychiatr. Scand..

[9] Flanagan E.W., Beyl R.A., Fearnbach S.N., Altazan A.D., Martin C.K., Redman L.M. (2021). The impact of covid-19 stay-at-home orders on health behaviors in adults. Obesity.

[10] Pellegrini M., Ponzo V., Rosato R., Scumaci E., Goitre I., Benso A., Belcastro S., Crespi C., De Michieli F., Ghigo E. (2020). Changes in weight and nutritional habits in adults with obesity during the “lockdown” period caused by the covid-19 virus emergency. Nutrients.

[11] OCDE (2019). The Heavy Burden of Obesity.

[12] Falagas M.E., Kompoti M. (2006). Obesity and infection. Lancet Infect. Dis..

[13] Calle E.E., Thun M.J., Petrelli J.M., Rodriguez C., Heath C.W. (1999). Body-mass index and mortality in a prospective cohort of U.S. Adults. N. Engl. J. Med..

[14] Chang T.H., Chou C.C., Chang L.Y. (2020). Effect of obesity and body mass index on coronavirus disease 2019 severity: A systematic review and meta-analysis. Obes. Rev..

[15] Simonnet A., Chetboun M., Poissy J., Raverdy V., Noulette J., Duhamel A., Labreuche J., Mathieu D., Pattou F., Jourdain M. (2020). High prevalence of obesity in severe acute respiratory syndrome coronavirus-2 (sars-cov-2) requiring invasive mechanical ventilation. Obesity.

[16] Bousquet J., Anto J.M., Iaccarino G., Czarlewski W., Haahtela T., Anto A., Akdis C.A., Blain H., Canonica G.W., Cardona V. (2020). Is diet partly responsible for differences in covid-19 death rates between and within countries?. Clin. Transl. Allergy.

[17] Khan M.A., Moverley Smith J.E. (2020). “Covibesity”, a new pandemic. Obes. Med..

[18] Thorning T.K., Fabre O., Legrand R., Astrup A., Hjorth M.F. (2018). Weight loss and weight loss maintenance efficacy of a novel weight loss program: The retrospective rnpc^®^ cohort. OBMED Obes. Med..

[19] The Silent Pandemic: How Lockdown is Affecting Future Health. https://covid.joinzoe.com/post/lockdown-weight-gain.

[20] Sondage Ifop Pour Darwin Nutrition: L’impact du Confinement sur l’alimentation des Français.Es. https://www.darwin-nutrition.fr/actualites/alimentation-francais/.

[21] Tronieri J.S., Wadden T.A., Chao A.M., Tsai A.G. (2019). Primary care interventions for obesity: Review of the evidence. Curr. Obes. Rep..

[22] Byaruhanga J., Atorkey P., McLaughlin M., Brown A., Byrnes E., Paul C., Wiggers J., Tzelepis F. (2020). Effectiveness of individual real-time video counseling on smoking, nutrition, alcohol, physical activity, and obesity health risks: Systematic review. J. Med. Internet Res..

[23] Hutchesson M.J., Rollo M.E., Krukowski R., Ells L., Harvey J., Morgan P.J., Callister R., Plotnikoff R., Collins C.E. (2015). Ehealth interventions for the prevention and treatment of overweight and obesity in adults: A systematic review with meta-analysis. Obes. Rev..

[24] Huang J.W., Lin Y.Y., Wu N.Y. (2019). The effectiveness of telemedicine on body mass index: A systematic review and meta-analysis. J. Telemed. Telecare.

[25] Varkevisser R.D.M., van Stralen M.M., Kroeze W., Ket J.C.F., Steenhuis I.H.M. (2019). Determinants of weight loss maintenance: A systematic review. Obes. Rev..

[26] Car J., Koh G.C., Foong P.S., Wang C.J. (2020). Video consultations in primary and specialist care during the covid-19 pandemic and beyond. BMJ.

[27] Bhutani S., Cooper J.A. (2020). Covid-19-related home confinement in adults: Weight gain risks and opportunities. Obesity.

[28] Di Renzo L., Gualtieri P., Pivari F., Soldati L., Attina A., Cinelli G., Leggeri C., Caparello G., Barrea L., Scerbo F. (2020). Eating habits and lifestyle changes during covid-19 lockdown: An italian survey. J. Transl. Med..

