# Supplementary material for: The Impact of the COVID-19 Lockdown on Weight Loss and Body Composition in Subjects with Overweight and Obesity Participating in a Nationwide Weight-Loss Program: Impact of a Remote Consultation Follow-Up—The CO-RNPC Study
Source: Nutrients. 2021 Jun 23;13(7):2152. doi: 10.3390/nu13072152 (PMC8308256; doi:10.3390/nu13072152)
Supplement: Supplementary file 1 [file nutrients-13-02152-s001.zip › nutrients-1240752-supplementary.pdf]

eTable 1: Description of changes in body composition and rate of change before, during and after COVID19 lockdown periods

	Period	Initial value (cm or %)	Final value (cm or %)	Delta (cm or %)	Pvalue*
Waist circumference (cm)	Before Lockdown	102.0 [93.0 ; 112.0]	101.0 [92.0 ; 111.0]	-1 [-2 ; 0]	<.01
	Lockdown	101.0 [92.0 ; 111.0]	98.0 [90.0 ; 107.0]	-2 [-6 ; 0]	<.01
	After lockdown	98.0 [90.0 ; 107.0]	98.0 [89.0 ; 107.0]	0 [-1 ; 0]	<.01
Fat mass (%)	Before Lockdown	40.6 [37.0 ; 44.4]	40.2 [36.6 ; 44.0]	-0.4 [-0.7 ; 0]	<.01
	Lockdown	40.2 [36.6 ; 44.0]	39.1 [35.5 ; 43.2]	-0.9 [-2.3 ; 0.2]	<.01
	After lockdown	39.1 [35.5 ; 43.2]	38.9 [35.2 ; 42.9]	-0.2 [-0.5 ; 0.1]	<.01
Muscle mass (%)	Before Lockdown	29.6 [27.5 ; 33.0]	29.7 [27.6 ; 33.1]	0.1 [0 ; 0.2]	<.01
	Lockdown	29.7 [27.6 ; 33.1]	30.0 [27.8 ; 33.7]	0.2 [-0.1 ; 0.7]	<.01
	After lockdown	30.0 [27.8 ; 33.7]	30.1 [27.9 ; 33.8]	0 [-0.1 ; 0.2]	<.01
Body water (%)	Before Lockdown	43.1 [40.4 ; 45.8]	43.5 [40.7 ; 46.3]	0.2 [0 ; 0.5]	<.01
	Lockdown	43.5 [40.7 ; 46.3]	44.2 [41.3 ; 46.9]	0.6 [-0.2 ; 1.6]	<.01
	After lockdown	44.2 [41.3 ; 46.9]	44.4 [41.4 ; 47.1]	0.1 [-0.1 ; 0.4]	<.01

Median period durations between two measures: before lockdown: 15 days [15; 17], during LD: 99 days [92; 106], after LD: 15 days [15; 21]. Values are presented in median and interquartile range

*All p values were results of paired t-test comparing initial and final weights

eTable 2: Description of changes in body composition and rate of change before, during and after COVID19 lockdown periods for women (N=1,246)

	Period	Initial value (cm or %)	Final value (cm or %)	Delta (cm or %)	Pvalue*
Waist circumference (cm)	Before Lockdown	100 [91 ; 108]	98 [90 ; 107]	-1 [-2 ; 0]	<·01
	Lockdown	98 [90 ; 107]	96 [88 ; 104]	-2 [-6 ; 0]	<·01
	After lockdown	96 [88 ; 104]	95 [88 ; 103]	0 [-1 ; 0]	<·01
Fat mass (%)	Before Lockdown	41·5 [38·2 ; 45·0]	41·1 [37·8 ; 44·7]	-0·4 [-0·7 ; 0·0]	<·01
	Lockdown	41·1 [37·8 ; 44·7]	40·0 [36·5 ; 43·7]	-0·9 [-2·3 ; 0·2]	<·01
	After lockdown	40·0 [36·5 ; 43·7]	39·8 [36·2 ; 43·5]	-0·2 [-0·5 ; 0·2]	<·01
Muscle mass (%)	Before Lockdown	28·9 [26·8 ; 31·2]	29·0 [27·0 ; 31·3]	0·1 [0·0 ; 0·2]	<·01
	Lockdown	29·0 [27·0 ; 31·3]	29·2 [27·3 ; 31·7]	0·2 [-0·1 ; 0·6]	<·01
	After lockdown	29·2 [27·3 ; 31·7]	29·2 [27·4 ; 31·7]	0·0 [-0·1 ; 0·1]	<·01
Body water (%)	Before Lockdown	42·5 [39·9 ; 45·0]	42·7 [40·1 ; 45·4]	0·2 [0·0 ; 0·5]	<·01
	Lockdown	42·7 [40·1 ; 45·4]	43·5 [40·7 ; 46·1]	0·6 [-0·2 ; 1·6]	<·01
	After lockdown	43·5 [40·7 ; 46·1]	43·6 [40·9 ; 46·3]	0·1 [-0·1 ; 0·4]	<·01

Values are presented in median and interquartile range·

*All p values were results of paired t-test comparing initial and final weights

eTable 3: Description of changes in body composition and rate of change before, during and after COVID19 lockdown periods for men (N=304)

	Period	Initial value (cm or %)	Final value (cm or %)	Delta (cm or %)	Pvalue*
Waist circumference (cm)	Before Lockdown	100 [91 ; 108]	98 [90 ; 107]	-1 [-2 ; 0]	<·01
	Lockdown	98 [90 ; 107]	96 [88 ; 104]	-2 [-6 ; 0]	<·01
	After lockdown	96 [88 ; 104]	95 [88 ; 103]	0 [-1 ; 0]	<·01
Fat mass (%)	Before Lockdown	36·3 [33·1 ; 40·6]	36·0 [32·7 ; 40·1]	-0·3 [-0·8 ; 0·0]	<·01
	Lockdown	36·0 [32·7 ; 40·1]	35·2 [31·2 ; 38·5]	-0·8 [-2·3 ; 0·2]	<·01
	After lockdown	35·2 [31·2 ; 38·5]	34·9 [31·1 ; 38·3]	-0·2 [-0·6 ; 0·1]	<·01
Muscle mass (%)	Before Lockdown	34·5 [31·2 ; 36·5]	34·6 [31·4 ; 36·8]	0·2 [0·0 ; 0·4]	<·01
	Lockdown	34·6 [31·4 ; 36·8]	35·1 [32·3 ; 37·3]	0·4 [-0·1 ; 1·3]	<·01
	After lockdown	35·1 [32·3 ; 37·3]	35·1 [32·3 ; 37·5]	0·1 [-0·1 ; 0·3]	<·01
Body water (%)	Before Lockdown	46·2 [43·4 ; 48·8]	46·6 [43·7 ; 49·0]	0·2 [0·0 ; 0·5]	<·01
	Lockdown	46·6 [43·7 ; 49·0]	47·1 [44·6 ; 49·8]	0·6 [-0·2 ; 1·7]	<·01
	After lockdown	47·1 [44·6 ; 49·8]	47·2 [44·9 ; 49·8]	0·1 [-0·1 ; 0·4]	<·01

Values are presented in median and interquartile range·

*All p values were results of paired t-test comparing initial and final weights

Table 4: Comparison of weight loss and rate of weight loss according to participation in remote consultation follow-up

Variables	No remote consultation (N=265)	Remote consultation (N=1,285)	Missing values (N)	Pvalue
**Before lockdown**				
Delta weight loss (kg)	-1.0 [-1.7 ; -0.2]	-1.2 [-2.1 ; -0.3]	0	0.01
Delta weight loss (%)	-1.1 [-2 ; -0.2]	-1.3 [-2.3 ; -0.4]	0	0.02
Rate of weight loss (kg/week)	-0.4 [-0.8 ; -0.1]	-0.6 [-1 ; -0.2]	6	<.01
**During lockdown**				
Delta weight loss during lockdown (kg)	-0.7 [-3.8 ; 1.6]	-2.9 [-6.7 ; 0.2]	0	<.01
Delta weight loss during lockdown (%)	-0.8 [-4.5 ; 2.1]	-3.4 [-7.6 ; 0.2]	0	<.01
Rate of weight loss (kg/week)	-0.1 [-0.1 ; 0]	-0.1 [-0.1 ; 0]	0	0.05
**After lockdown**				
Delta weight loss (kg)	-0.4 [-1 ; 0.3]	-0.5 [-1.2 ; 0.2]	0	0.02
Delta weight loss (%)	-0.4 [-1.1 ; 0.5]	-0.6 [-1.5 ; 0.2]	0	<.01
Rate of weight loss (kg/week)	-0.2 [-0.4 ; 0.2]	-0.2 [-0.6 ; 0.1]	6	<.01

Median period durations between two measures: before lockdown: 15 days [15; 17], during LD: 99 days [92; 106], after LD: 15 days [15; 21].

eFigure 1: Rates of change in body composition before, during and after COVID19 lockdown

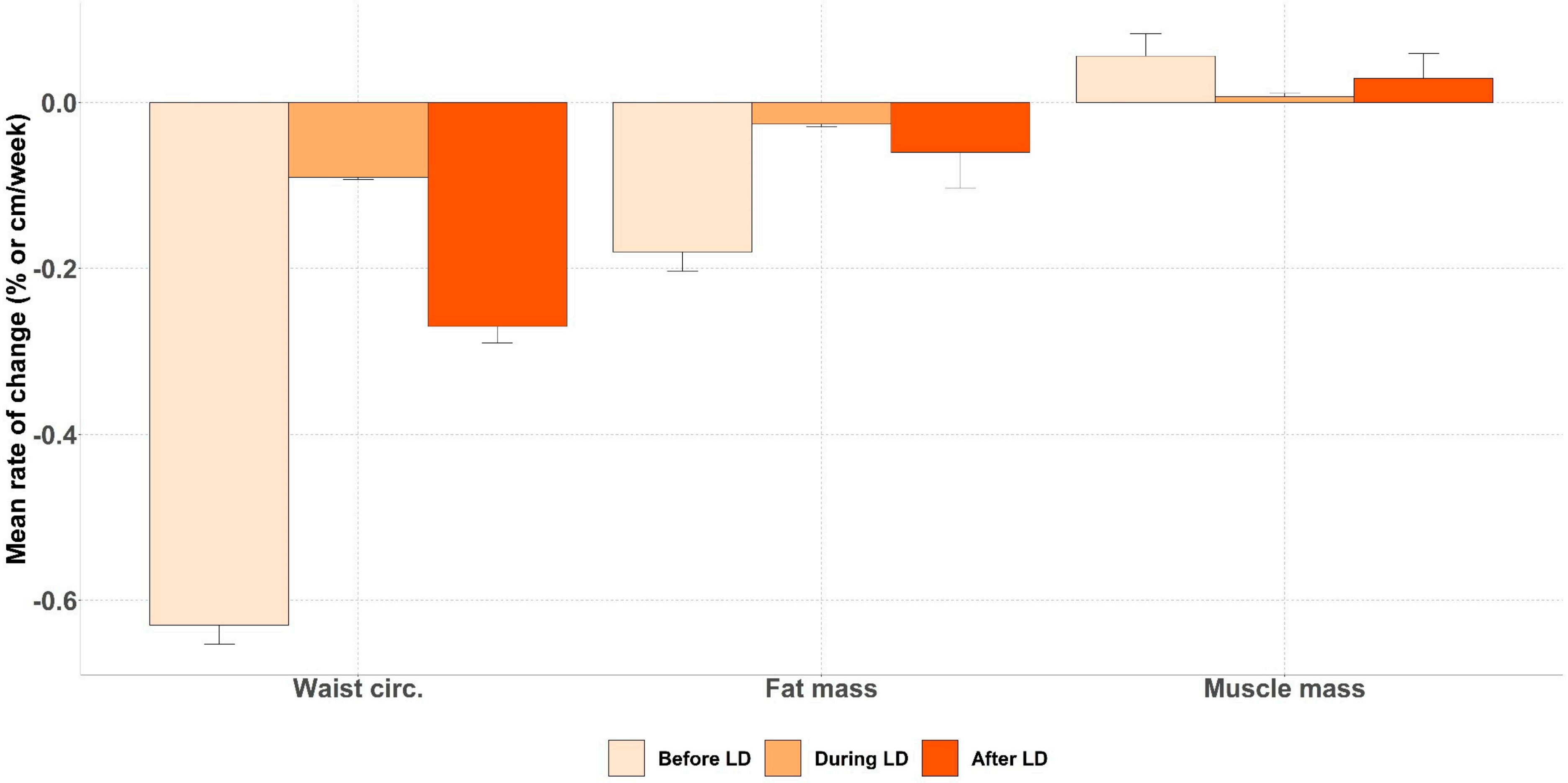

LD: lockdown; Waist circ.: waist circumference
